# Transbronchial drainage using endobronchial ultrasonography with guide sheath for lung abscess: A case report

**DOI:** 10.1097/MD.0000000000010812

**Published:** 2018-05-18

**Authors:** Daizo Yaguchi, Motoshi Ichikawa, Noriko Inoue, Daisuke Kobayashi, Masato Shizu, Naoyuki Imai

**Affiliations:** Department of Respiratory Medicine, Gifu Prefectural Tajimi Hospital, Tajimi, Gifu, Japan.

**Keywords:** EBUS-GS, lung abscess, transbronchial drainage

## Abstract

**Rationale:**

Lung abscess was previously treated surgically, but is now mainly treated with antibiotics and ideally with direct drainage, although postural drainage canalso be used.

**Patient concerns:**

A chest abnormal shadow was detected in an 82-year-old man and he was referred to our department in November 2017. On chest computed tomography (CT), a low-density mass shadow was present in the left S8 segment. Lung abscess and lung cancer were considered as differential diagnoses, and treatment with sulbactam sodium/ampicillin sodium (SBT/ABPC) was first initiated for lung abscess. The etiologic agent could not be identified by sputum examination, and the abscess shadow remained.

**Diagnoses:**

Lung abscess.

**Interventions:**

Endobronchial ultrasonography with a guide sheath (EBUS-GS)-guided bronchoscopy was performed on hospital day 21 to diagnose the lesion, identify the etiologic agent if the lesion was a lung abscess, and attempt drainage. Vacuum aspiration performed in the guide sheath after the probe was placed within the lesion produced 4-5 ml of gray turbid pus, and the abscess was judged to have been drained.

**Outcomes:**

A subsequent pathological examination did not detect malignant cells. *Klebsiella pneumoniae*, *Prevotella* spp. was identified as the etiologic agent in bacteriological tests. Antibiotics were changed based on sensitivity test results, and drainage was similarly performed on hospital day 28. The shadow gradually improved and disappeared. Therefore, this procedure and treatment led to identification of the etiologic agent and helped with cure of the disease.

**Lessons:**

Based on the basic principle of treatment for abscess using as much drainage as possible, EBUS-GS-guided transbronchial drainage may be considered to be a “new procedure” for lung abscess.

## 1 Introduction

Lung abscess is mainly treated with antibiotics, but the effect is often insufficient and additional treatment is required in many cases.^[1,2]^ Direct and safe treatment of a lung abscess with drainage using endobronchial ultrasonography with a guide sheath (EBUS-GS) was reported by Kurimoto et al.^[3]^ This procedure has the aim of increasing the success rate of bronchoscopic drainage as an optional treatment, in addition to antibiotics, in a patient with a suspected lung abscess. As per our institution review board's policy, ethical approval was not necessary for the case report. Informed consent for publication was given by the patient.

## 2 Case report

**Figure 1:**
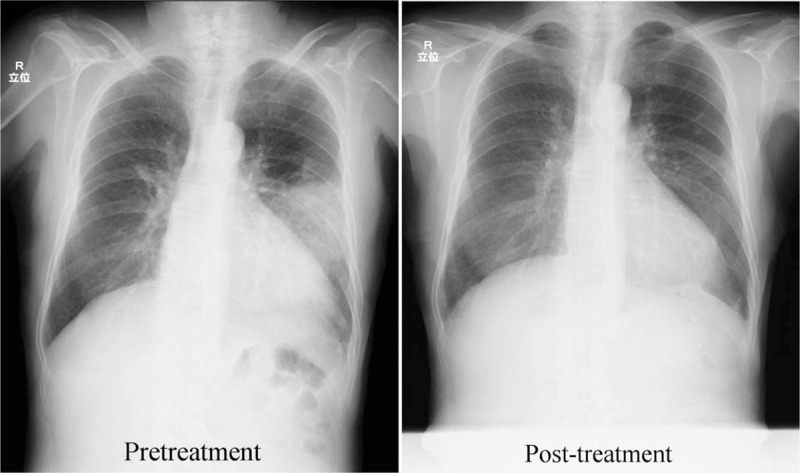
Chest x-ray. (A) On admission, a shadow was noted in the left middle over the lower lung field. (B) After treatment, the shadow was mostly resolved.

**Figure 2:**
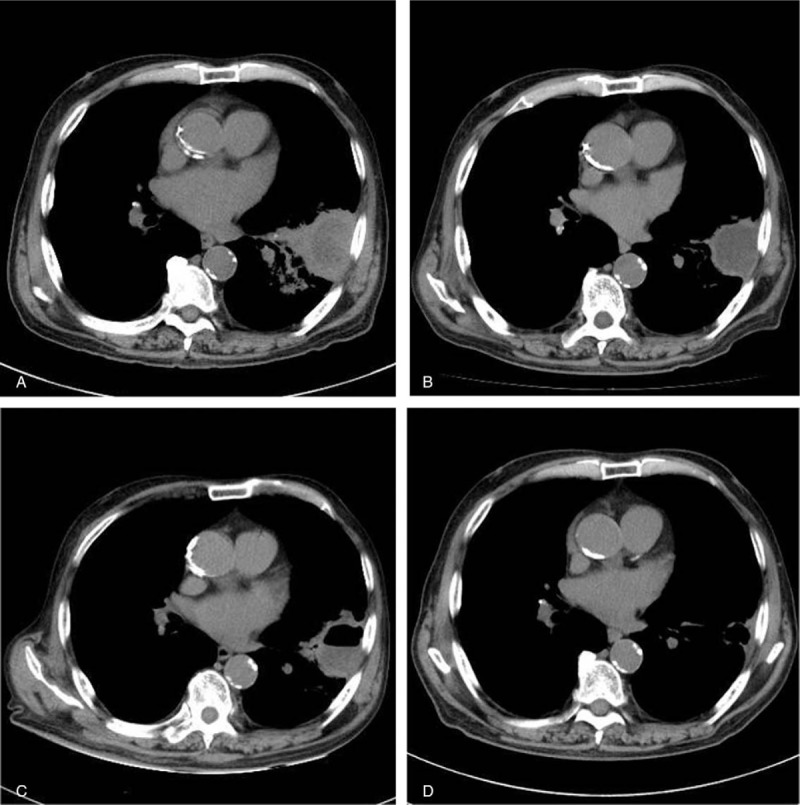
Chest CT (mediastinal window). (A) Before treatment, a 4-cm mass shadow with low-density content and a thick wall accompanied by surrounding inflammation was noted in the left lung field. (B) On hospital day 20, the wall of the mass shadow thinned and the surrounding inflammation remitted, but the low-density content remained. (C) Immediately after EBUS-GS-guided transbronchial drainage on hospital day 21, the content of the mass observed on hospital day 20 was drained, resulting in a cavity. (D) On hospital day 56, the mass had disappeared and only a slight shadow remained.

The patient was an 82-year-old man with chief complaints of left chest pain and cough. His medical history included coronary artery stenosis (stenting). He was an ex-smoker and he was currently being treated with two oral antithrombotic drugs. He had visited a physician for left chest pain and cough that had persisted for several days. Features of pneumonia were observed on chest plain radiography, and the patient was referred to our hospital. Blood tests showed C-reactive protein level 22 mg/dL, lactate dehydrogenase level 214 U/L, serum carcinoembryonic antigen 1.96 ng/mL, and cytokeratin fragment 1.9 ng/mL. In imaging findings (Figs. 1 and 2), chest x-ray showed a shadow in the left middle over the lower lung field, and computed tomography (CT) showed a 4-cm mass shadow with low-density content in the left S8 segment. Mediastinal lymph node swelling was not noticeable.

**Figure 3:**
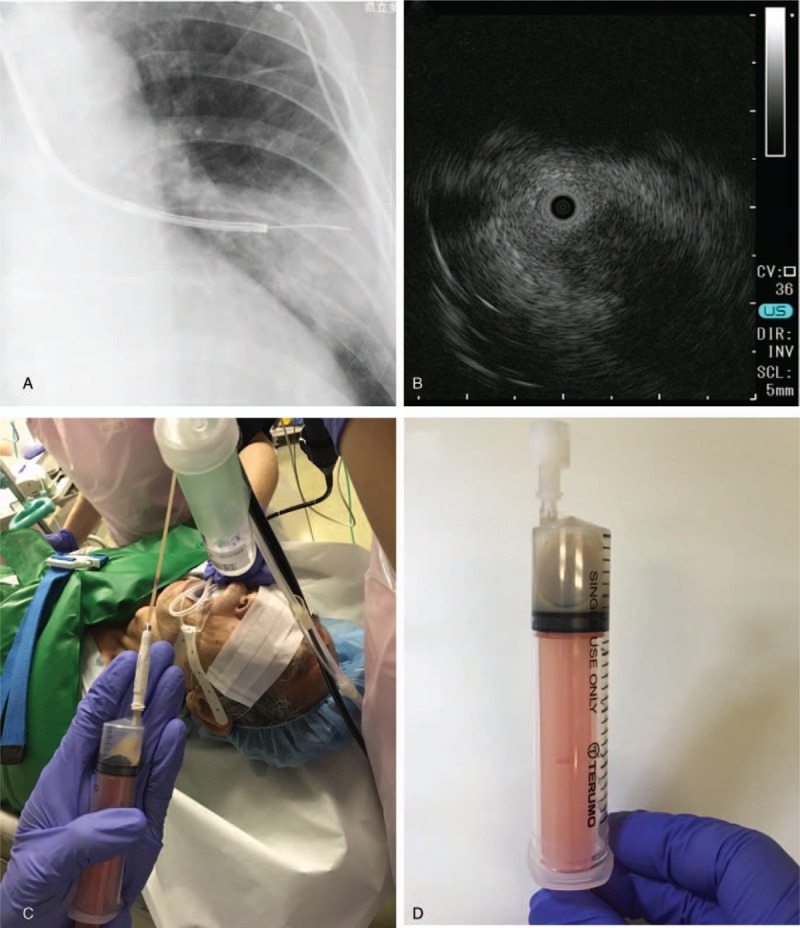
(A) Fluoroscopic image of the guide sheath placed in the shadow. (B) Ultrasound imaging in which the probe was placed within the lesion using radial EBUS. (C) Pus being collected into a syringe through the guide sheath during bronchoscopy. (D) A total of 4–5 mL of gray turbid pus was collected.

On chest CT, lung abscess and lung cancer were considered as differential diagnoses. Antibiotic treatment with SBT/ABPC was initiated for suspected lung abscess, but the etiologic agent could not be identified by sputum examination and the abscess shadow remained on imaging. Bronchoscopy was scheduled after about one week, but since the patient was being treated with oral antithrombotic drugs, a washout period was required. Therefore, endobronchial ultrasonography with EBUS-GS-guided bronchoscopy was finally performed on hospital day 21 to diagnose the lesion, identify the etiologic agent if the lesion was a lung abscess, and attempt drainage. After EBUS images confirmed that the probe was located within the lesion, vacuum aspiration using an empty injector to the guide sheath (ϕ1.95 mm) resulted in collection of 4 to 5 mL of gray turbid pus, indicating that the abscess was drained (Fig. 3). On CT immediately after drainage, the drained region was observed as a cavity (Fig. 2). On subsequent pathological examination, no malignant cells were detected. *Klebsiella pneumoniae*, *Prevotella* spp. was identified as the etiologic agent in bacteriological tests. Antibiotics were changed based on sensitivity test results. Drainage was similarly performed on hospital day 28, and 3 to 4 mL of pus was drained. The shadow gradually improved and then disappeared (Figs. 1 and 2).

## 3 Discussion

Lung abscess was previously treated surgically using lung lobectomy, but is now mainly treated with antibiotics. Postural drainage is also considered,^[1,2]^ but the treatment effect is often insufficient. Direct drainage is ideal as a basic principle of treatment of abscess disease, and bronchoscopic transbronchial drainage has been reported for this purpose,^[4–8]^ but has not been established as a treatment method. Drainage by conventional transbronchial catheterization without concomitant use of EBUS (blind drainage) may fail because of the lack of reliability of catheterization into the abscess, and there is a need to devise a method to increase the success rate. Percutaneous drainage has also been reported,^[1,9–11]^ but has risks of empyema and pneumothorax caused by spread of bacteria into the thoracic cavity because of performance of the percutaneous approach via the pleura.

The main advantage of the EBUS-GS treatment is that the procedure can be rapidly switched to drainage after reliable detection of the lesion under EBUS-GS, although fluoroscopy is concomitantly used. If pus is drained through the guide sheath by removing the echo probe after the probe is placed within the lesion using radial EBUS and negative pressure aspiration of the contents of the guide sheath, drainage is successful. Differentiation from lung cancer on imaging is problematic in diagnosis of lung abscess, but drainage of pus under EBUS-GS increases the possibility that the disease is lung abscess. Therefore, when lung abscess and lung cancer are suspected on imaging, this procedure should be performed early, and when pus is drained under EBUS-GS, drainage treatment should be continued. Alternatively, if pus is not drained, the procedure can be switched to transbronchial biopsy (TBB) using EBUS-GS to examine the possibility of lung cancer.

EBUS-GS-guided transbronchial drainage has been found to be applicable and effective for an iatrogenic lung abscess formed as a complication after bronchoscopic lung biopsy,^[12]^ similarly to the present treatment, but the guide sheath was easily placed through the bronchial pathway used for biopsy because the procedure was performed after TBB. Our case illustrates that the procedure can be applied in general treatment for community-acquired lung abscess.

Homogenous density in the abscess on CT may be a pretreatment factor associated with successful completion of EBUS-GS-guided transbronchial drainage. Conversely, a thin guide sheath (ϕ1.95 mm) may lead to unsuccessful treatment because drainage may not be possible, even if the guide sheath is placed in the abscess lumen, depending on the properties of the abscess. Therefore, a thick guide sheath (ϕ2.55 mm) should be used when possible. Moreover, if a large part of the abscess shadow is a cavity, an image may not be acquired using EBUS, and the drainage effect may not be obtained, even though the guide sheath is placed correctly. However, when the guide sheath is placed in the cavity, saline can be injected and some effect may be obtained by irrigating the abscess. Regarding other factors, if a bronchus leading to the abscess (bronchus sign) is absent on CT before treatment, it may be difficult to select a bronchus in which to place the guide sheath, resulting in an unsuccessful outcome.

EBUS-GS-guided transbronchial drainage not only leads to the treatment of abscess disease, but also allows selection of appropriate antibiotics based on identification of the etiologic agent and a sensitivity test. This is an advantage of this treatment. In our patient, SBT/ABPC was administered as initial treatment and after our procedure, *K pneumoniae*, *Prevotella* spp was identified. *K pneumoniae* has only intermediate sensitivity to this drug, and treatment was switched to antibiotics to which the bacteria had greater susceptiblity. This may have contributed to shortening of the treatment period. Finally, good safety of EBUS-GS-guided bronchial biopsy has been reported in several studies,^[13,14]^ which suggests that EBUS-GS-guided transbronchial drainage may also be useful and safe.

## 4 Conclusion

EBUS-GS-guided transbronchial drainage may enable lung abscess drainage without complication. Based on the basic principle of treatment for abscess disease of as much drainage as possible, this approach may be considered to be a “new procedure” for lung abscess.

## Author contributions

**Conceptualization:** Daizo Yaguchi.

**Data curation:** Daizo Yaguchi.

**Formal analysis:** Daizo Yaguchi.

**Investigation:** Daizo Yaguchi.

**Methodology:** Daizo Yaguchi.

**Project administration:** Masato Shizu, Noriko Inoue, Daisuke Kobayashi, Naoyuki Imai.

**Resources:** Daizo Yaguchi.

**Validation:** Daizo Yaguchi.

**Visualization:** Daizo Yaguchi.

**Writing – original draft:** Daizo Yaguchi.

**Writing – review & editing:** Daizo Yaguchi, Motoshi Ichikawa.
